# The UNITE database for molecular identification of fungi: handling dark taxa and parallel taxonomic classifications

**DOI:** 10.1093/nar/gky1022

**Published:** 2018-10-29

**Authors:** Rolf Henrik Nilsson, Karl-Henrik Larsson, Andy F S Taylor, Johan Bengtsson-Palme, Thomas S Jeppesen, Dmitry Schigel, Peter Kennedy, Kathryn Picard, Frank Oliver Glöckner, Leho Tedersoo, Irja Saar, Urmas Kõljalg, Kessy Abarenkov

**Affiliations:** 1 University of Gothenburg, Department of Biological and Environmental Sciences, Gothenburg Global Biodiversity Centre, Box 461, 405 30 Gothenburg, Sweden; 2 Natural History Museum, University of Oslo, P.O. Box 1172, Blindern, 0318 Oslo, Norway; 3 The James Hutton Institute and University of Aberdeen, Aberdeen, UK; 4 Wisconsin Institute for Discovery, University of Wisconsin-Madison, 330 North Orchard Street, Madison, WI 53715, USA; 5 Centre for Antibiotic Resistance research (CARe) at University of Gothenburg, Gothenburg, Sweden; 6 Department of Infectious Diseases, Sahlgrenska Academy, University of Gothenburg, Guldhedsgatan 10, SE-413 46 Gothenburg, Sweden; 7 Global Biodiversity Information Facility, Universitetsparken 15, DK-2100 Copenhagen Ø, Denmark; 8 Department of Plant and Microbial Biology, University of Minnesota, 1479 Gortner Avenue, St. Paul, MN 55108, USA; 9 Department of Botany, National Museum of Natural History, Smithsonian Institution, Washington, DC 20013, USA; 10 Jacobs University Bremen and MPI for Marine Microbiology, Celsiusstr. 1, D-28359 Bremen, Germany; 11 University of Tartu, Institute of Ecology and Earth Sciences, 40 Lai Street, 51005 Tartu, Estonia; 12 Natural History Museum and Botanical Garden, University of Tartu, 46 Vanemuise Street, 51003 Tartu, Estonia

## Abstract

UNITE (https://unite.ut.ee/) is a web-based database and sequence management environment for the molecular identification of fungi. It targets the formal fungal barcode—the nuclear ribosomal internal transcribed spacer (ITS) region—and offers all ∼1 000 000 public fungal ITS sequences for reference. These are clustered into ∼459 000 species hypotheses and assigned digital object identifiers (DOIs) to promote unambiguous reference across studies. In-house and web-based third-party sequence curation and annotation have resulted in more than 275 000 improvements to the data over the past 15 years. UNITE serves as a data provider for a range of metabarcoding software pipelines and regularly exchanges data with all major fungal sequence databases and other community resources. Recent improvements include redesigned handling of unclassifiable species hypotheses, integration with the taxonomic backbone of the Global Biodiversity Information Facility, and support for an unlimited number of parallel taxonomic classification systems.

## INTRODUCTION

The fungal kingdom comprises an estimated 2.2–3.8 million species of heterotrophic eukaryotes, most of which are inconspicuous and substrate-dwelling (1). Molecular (DNA sequence) data are routinely used to explore fungi and fungal communities through barcoding and metabarcoding efforts. The ∼600-base nuclear ribosomal internal transcribed spacer (ITS) region is the primary genetic marker for such pursuits (2), and more than 1 000 000 full-length, Sanger-derived fungal ITS sequences are available for reference in the International Nucleotide Sequence Databases Collaboration (INSDC; 3). Significant processing and annotation are necessary before the public sequences can be used for taxonomic annotation of newly generated sequence data, and the UNITE database for molecular identification of fungi (4; https://unite.ut.ee/) was launched in 2003 as a curated copy of the public fungal ITS sequences. UNITE seeks to provide reproducible identification of fungi and facilitate mycological progress by assembling and disseminating taxonomic, ecological, and geographical metadata for all fungi known from ITS sequence data.

Fungal metabarcoding struggles with very large numbers of operational taxonomic units (OTUs; 5) that cannot be identified to any meaningful taxonomic lineage beyond, e.g. the kingdom or phylum level (6). UNITE regularly clusters all ITS sequences at several sequence similarity thresholds to obtain approximate species-level OTUs referred to as species hypotheses (SHs). All such SHs (458 797 as of August 2018) are assigned a unique digital object identifier (DOI) to allow stable, unambiguous reference across studies, even in the complete absence of meaningful taxonomic names. A designated URL (e.g. https://unite.ut.ee/bl_forw_sh.php?sh_name=SH181628.07FU) where all pertinent metadata are displayed is available for all SHs. Users can download the corresponding multiple sequence alignment files or compare newly generated sequences to the SHs through BLAST (7), the recently launched probabilistic assigner PROTAX (8), and other search and query functions. In addition, compatible datasets assembled from the SHs are provided for a range of metabarcoding software pipelines and other resources (https://unite.ut.ee/repository.php), notably QIIME (9), MOTHUR (10), USEARCH (11) and micca (12).

UNITE supports web-based third-party annotation of sequence data to reflect recent nomenclatural and taxonomic changes and to correct the often-suboptimal state of taxonomic annotation and other metadata items among public DNA sequences. Participation in sequence annotation efforts is encouraged through organization of annotation jamborees targeting, e.g. plant pathogenic fungi (13) or the built mycobiome (14). Any changes contributed are credited through co-authorship of the underlying DOIs, with citations to all DOIs (SHs) being monitored by DataCite (https://www.datacite.org/). The community-oriented nature of UNITE encourages participation but also incorporates a need for a review-type procedure of handling user-provided annotations. Such a system is implemented together with a range of other quality-control measures to maintain a high quality standard for the entries in UNITE.

The year 2003 saw the first public release of UNITE. Since then, mycology has undergone far-reaching changes in the wake of high-throughput sequencing (HTS) methods and the realisation that undescribed, ‘dark’ taxa permeate the fungal tree of life and may dominate the functional biodiversity of the planet. Changes in nomenclatural rules and taxonomic principles have further fuelled the field (15). In this paper, we detail the recent changes we have implemented in UNITE to meet the challenges posed by technological and conceptual advances in the mycological and molecular ecology communities.

## DATABASES

### Sequence data and quality control

Public fungal ITS sequences are sourced from the INDSC and subjected to a range of quality control measures, including processing with ITSx (16) and UCHIME (17) in an attempt to reject non-ITS and chimeric sequences. Substandard entries are kept for future reference but are not used for identification purposes. Sequences found to contain the full ITS2 subregion of the ITS region are clustered at 97–100% similarity in steps of 0.5% to produce SHs. A representative sequence is chosen randomly from the most abundant sequence type in each SH, although this choice can be overridden manually to reflect, e.g. sequences derived from type material. Efforts are under way to also include partial ITS sequences stemming from HTS studies in this system. The mind-boggling number of such sequences (>1 billion partial fungal ITS reads in Sequence Read Archive (18,19)) coupled with the difficulties of associating non-overlapping datasets of ITS1 and ITS2 sequences impede progress. We are currently working on adding ITS2-derived HTS studies to UNITE. In parallel, long HTS reads from technologies such as PacBIO (https://www.pacb.com/) and Oxford Nanopore (https://nanoporetech.com/) are gradually becoming available. We have added a first set of long PacBio reads comprising the full ITS region and some 1000 bases of the LSU gene to UNITE and the species hypothesis system.

Web-based third-party annotation of sequence entries covers most aspects of public sequences, including taxonomic name, country of collection, and substrate of collection. A history of all annotations is kept so that it is possible to track changes over time, e.g. in the names given to a sequence. This allows for competing views of taxonomic annotations to be expressed. As of fall 2018, a total of 276 889 third-party annotations have been provided by UNITE users (including 101 833 additions of country of collection, 69 539 annotations of substrate of collection and 23 410 taxonomic re-annotations).

### UNITE taxonomy

By default, UNITE uses the NCBI Taxonomy classification (20) as taxonomic backbone, supplemented with modifications from Index Fungorum (http://www.indexfungorum.org) and MycoBank (21). A breakdown of the SHs in terms of taxonomy and geography (August 2018) is provided in Table 1. UNITE has the ambition to offer all public fungal ITS sequences to the user, and these data are often complicated from a taxonomic point of view. Taxonomic misidentification is rife, as are sequences lacking meaningful taxonomic annotation in the first place (e.g. ‘Uncultured fungus’). SHs with sequences of conflicting taxonomic information are flagged for manual curation by curators or experienced users. Such trusted third-party users can rename sequences through their web browser. Sequences from type material (e.g. GenBank RefSeq Loci; 22) are used to inform the taxonomic annotation of similar sequences. For instance, an unnamed sequence that is at least 97% similar in a global ITS alignment to a fully annotated type-derived sequence can safely be annotated at the family and typically also at the genus levels. The original name as well as the history of re-annotations are kept for reference for all sequences.

**Table 1.** A breakdown of the SHs in UNITE (August 2018) in terms of taxonomy (following 23) and geography, using the 98.5% threshold level.

Subkingdom	Number of SHs (at 1.5% threshold)
Dikarya (Basidiomycota + Ascomycota)	63 062
Unidentified	6071
Mucoromyceta	3711
Chytridiomyceta	502
Rozellomyceta	200
Zoopagomyceta	76
Blastocladiomyceta	42
Basidiobolomyceta	22
Olpidiomyceta	14
Aphelidiomyceta	5
GS01	2
Country	Number of SHs (at 1.5% threshold)
United States	13 802
China	7502
Canada	4191
Japan	3883
Germany	3667
Australia	3624
Spain	3065
Estonia	3047
Finland	3039
Sweden	2949

Most SHs comprise sequences from multiple countries, such that many SHs contributed to several countries in the geography list. The 10 countries with the largest number of SHs are shown.

There is no generally agreed upon, up-to-date system for fungal classification, although a synthesis was recently published (23). Fungal classification is in a state of flux, and different, partly incompatible classification systems are used across mycological resources such as Index Fungorum (http://www.indexfungorum.org), MycoBank (http://www.mycobank.org) and the INDSC (24). To address the need to navigate across competing taxonomic systems, the new release of UNITE allows representation of an arbitrary number of alternative taxonomic systems. For this we introduce the Taxon Hypothesis (TH) concept that allows communication of sequence-based SHs over many classifications at the same time. As with SHs, all THs will receive unique DOI-based stable identifiers, and single SHs can belong to different higher taxa in different classifications. The results of different studies are comparable and re-usable when TH DOIs are used for communication. The taxon hypothesis pages are due for launch late 2018.

### Database structure and adherence to metadata standards

UNITE is an assemblage of datasets managed on the PlutoF platform (https://plutof.ut.ee/), which uses PostgreSQL (https://www.postgresql.org/) and the PostGIS database engines as well as the Ember.js and DRF frameworks (25). PlutoF comprises nearly 200 tables and is modelled to allow exact, standards-compliant representation of DNA sequences and sequence metadata in terms of, e.g. taxonomy, nomenclature, ecology, and geography. For sequence data and metadata, the MIxS standard (26) is implemented. For the taxonomical, ecological, and other data types, a range of standards is used (e.g. DarwinCore, Ecological Metadata Language, and the Microbiological Common Language). The PlutoF platform is a part of the DataCite consortium, which allows UNITE to publish SHs with DOIs. The PlutoF platform collaborates with the Global Biodiversity Information Facility (GBIF; https://www.gbif.org/), and sequence-based SHs from UNITE are as of June 2018 part of the GBIF taxonomic backbone. This makes it possible to upload the results of metabarcoding studies directly into the GBIF database (https://www.gbif.org/news/2LrgV5t3ZuGeU2WIymSEuk) and have them scored as biological observations even if no formal scientific names are available for the underlying taxa.

### Datasets

#### UNITE core release

The sequence data of UNITE are available to the user in a number of interactive and static ways. Our non-redundant core release (https://unite.ut.ee/repository.php) comprises a representative sequence from each non-singleton SH and currently includes 458 797 SHs variously delimited at 97–100% similarity to reflect the species level as closely as possible in light of the differences in intraspecific ITS variability across the fungal tree of life (2). This release is provided in the FASTA format (27) for, e.g. local BLAST searches, as well as formats tailored for a range of metabarcoding software pipelines, including QIIME, MOTHUR and USEARCH. A generic FASTA release of all ∼1 000 000 sequences is also available. Experienced users will find additional variations of these files available, differing in whether singleton SHs are included or not, the way sequence trimming was carried out, and the way the taxonomic affiliation of the sequences is provided. All releases include taxonomic re-annotations, such that they differ significantly from the INSDC release of the same fungal ITS sequences.

#### UNITE auxiliary releases

UNITE provides a number of release datasets tailored to meet specific requests and needs of the scientific community (https://unite.ut.ee/repository.php). For example, the ‘top 50 most wanted species of fungi’ release comprises the largest SHs for which no meaningful taxonomic annotation at, e.g. the phylum level can be derived at present (6). Its purpose is to encourage the scientific community to clarify the taxonomic affiliation of the underlying taxa and to speed up their formal characterization and description (28). Owing to user requests, we also provide our data in, e.g. the JSON format. We are happy to consider requests for additional formats and data releases. UNITE is a LinkOut provider for GenBank, which links their full-length fungal ITS sequences to the corresponding SH pages in UNITE. Since 2018, UNITE is also a data and link provider for the Global Biodiversity Information Facility (https://www.gbif.org/). This allows sequence-based observations of fungi annotated against the UNITE SH system to be indexed by and queried through GBIF. SHs became the first system to allow for incorporation of molecularly detected and molecularly identified biodiversity data into GBIF′s global data pool, currently dominated by morphological evidence from natural history collections and citizen science projects. In addition to scientific use, GBIF-mediated data are used for biodiversity assessments in global policy making and conservation efforts.

#### Dataset exports by users

The fact that UNITE implements a range of scientific standards means that the user can tailor pretty much any imaginable query governed by those standards. For example, it is possible to download all sequences from the built environment or sequences collected above some specific altitude or in Swedish forests, owing to the MIxS-BE and other standards. Targeting sequences isolated from ferns, the genus *Eucalyptus* or aquatic environments is similarly straightforward, as is targeting pathogenic or human-associated fungi. Downloads can be made in a number of formats, including FASTA, comma-separated values, as well as user-specified exports.

## UNITE WEBSITE

The UNITE website (https://unite.ut.ee) provides direct access to all SHs, all release files, and all metadata. The data are made available under the CC BY-SA 4.0 software license. No registration or login is needed to download data or do basic metadata and sequence searches. However, to access the more advanced features of UNITE, registration is necessary. Registration is a tiered process, where basic registration opens up all data access and download features. Modifying or depositing data requires additional clearance, and a sophisticated system is in place to allow control of what specific users may and may not do. A review-type process operates to vet data and annotations provided by users. Registered users can apply to become curators of specific taxa (e.g. the family Cantharellaceae), which enables them to re-annotate sequences and provide new metadata for those taxa. Documented taxonomic expertise is needed to become a curator of specific groups.

### Species hypothesis system

**Figure 1.**
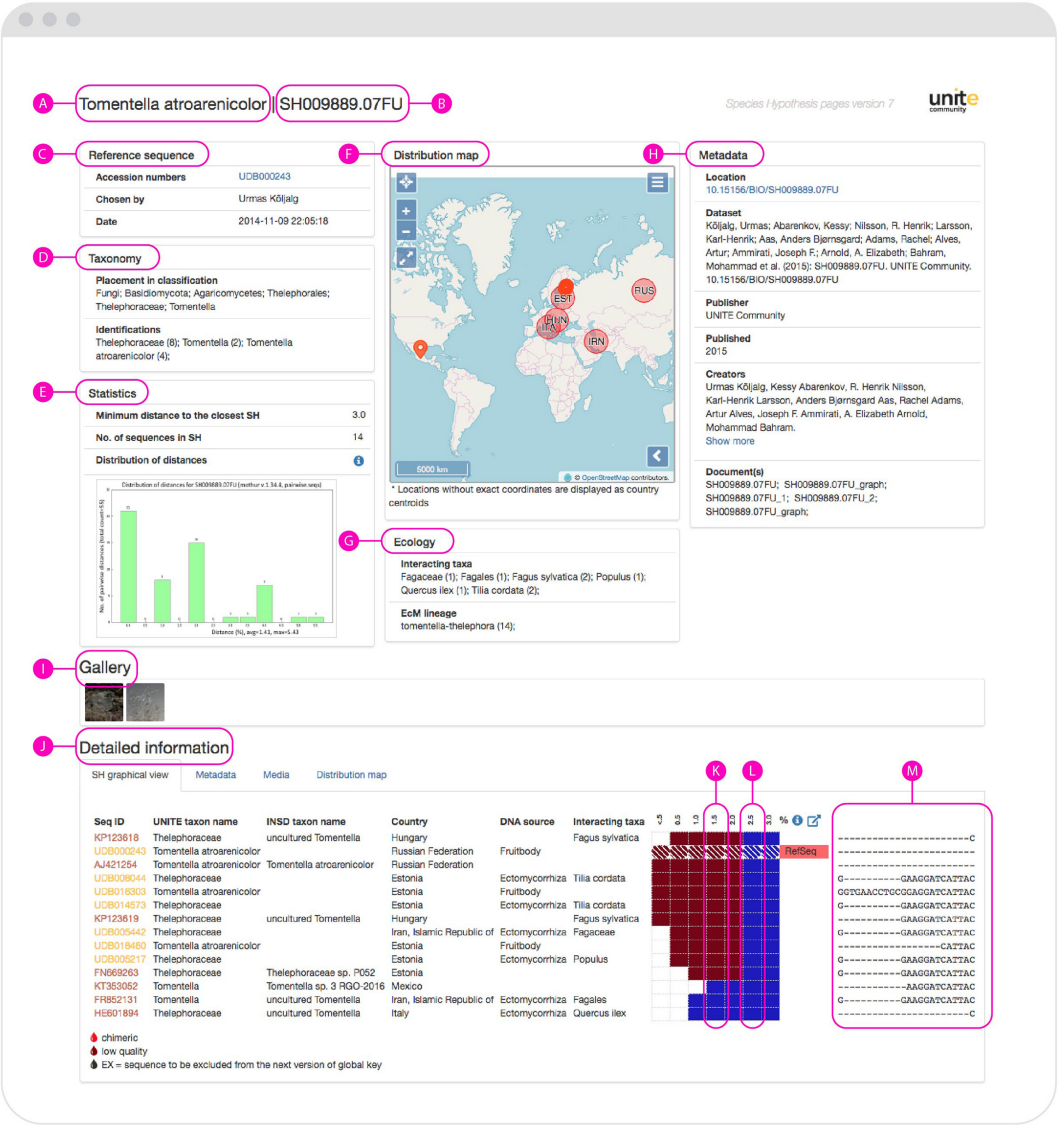
A screenshot of a UNITE SH Digital Object Identifier (DOI) page for *Tomentella atroarenicolor* (https://plutof.ut.ee/#/datacite/10.15156%2FBIO%2FSH009889.07FU). (**A**) The most accurate taxon name chosen automatically (or manually, if the default was overridden by an expert user) from the available sequence identifications. (**B**) Short ID of the DOI. (**C**) Data on reference sequence chosen to represent this SH. (**D**) Placement of the SH in the fungal classification and identification records for individual sequences. The number after the taxon name indicates how many sequences that carry that name. (**E**) Select statistics on the SH. The minimum distance 3.0% is the mandatory genetic difference between sister SHs. (**F**) Distribution map of the individual sequences. (**G**) Information on ecology (interacting taxa) if associated with the individual sequences. (**H**) DataCite-specific data on the DOI. (**I**) Images of the specimen or sample from which the DNA was extracted. Only a limited number of sequences have images attached to them. (**J**) Graphical overview of the SH with detailed information. (**K**) SH inclusiveness across sequence similarity threshold values. A threshold value ( = minimum distance) of 1.5% will split these sequences into two SHs, shown here in different colours. (**L**) A threshold value of 2.5% will lump all sequences into a single SH. Each such SH is hyperlinked to its own unique web page. (**M**) Scrollable multiple sequence alignment of the SH. ‘RefSeq’ indicates that the sequence was selected manually to be the representative sequence for the SHs. RefSeqs stem from type specimens or other authentic and particularly trustworthy material. This particular SH contains both INSDC sequences (brown) and sequences that are only found in UNITE (yellow). Some 29 000 sequences are only found in UNITE at this stage, but will be submitted to the INSDC upon publication of the underlying studies. These sequences are included in the various UNITE sequence releases and download files.

All SHs have a unique URL where the underlying multiple sequence alignments and pertinent metadata are displayed. The fact that each SH has a unique DOI makes them communicable across datasets and studies, even if an SH lacks a meaningful taxonomic annotation altogether (e.g. ‘Uncultured fungus’). UNITE employs the DOI versioning system, such that it is possible to track SH sequence inclusiveness over time. It is, thus, always possible to establish exactly what sequences were part of some particular SH at some specific point in time. We encourage users to explore their fungi of interest in our system, which brings together and visualizes sequences and their metadata in a way that the INSDC cannot (Figure 1).

### Identification services

Although UNITE is primarily a data provider rather than a metabarcoding or sequence analysis software pipeline, we do provide basic means for establishing the taxonomic affiliation of newly generated fungal ITS sequences. A multi-query BLAST service is provided, as is a function to assign sets of sequences to SHs. These searches enjoy the 23 000+ taxonomic re-annotations provided by the UNITE community, such that queries in UNITE offer information that is not present in the INSDC. All re-annotations are shown with original data, and although UNITE shares re-annotations with other sequence databases including the INSDC, many of them operate under policies that do not permit changing names or other conceptual aspects of sequences before obtaining written consent by the original sequence authors.

## OUTLOOK

The rapid development of single-molecule high-throughput sequencing technologies is enabling novel approaches to generating high-quality sequences spanning the full nuclear ribosomal operon, including the SSU, ITS and LSU markers in their entirety (29). We have begun collaborating and exchanging results with the SILVA database (30), which targets the prokaryotic and eukaryotic SSU and LSU genes but not the intercalary eukaryotic ITS region. The fairly conserved SSU and LSU genes offer the advantage of robust phylogenetic assignment of newly generated sequence data at the phylum, class, and order levels (and often further), however at the expense of resolution at the species and sometimes genus and family levels (31–32). Improved UNITE-SILVA collaboration would offer both robust phylogenetic placement and unambiguous communicability at the species level, a much sought-after combination (33).

UNITE will continue to dynamically provide versions of the SH taxonomies to the GBIF backbone taxonomy, and molecular occurrence data from specimens and metabarcoding samples will become discoverable through https://www.gbif.org as well. We hope and expect that extending the mapping of biodiversity into the molecular realm will lead to richer and unbiased biodiversity evidence to positively impact global modelling and decision-making.

UNITE seeks to provide resolved taxonomic information for fungal ITS sequences across the fungal tree of life, and employs several mechanisms to try to ensure that. However, the taxonomic placement infers only a part of the biology of an organism. Recently, we have begun to collaborate with the FUNGuild database (34) to provide information on the functional guild assignments of the fungal species and groups in UNITE. We will shortly be able to show not only taxonomic names, but also the functional guilds of those fungi (e.g. mycorrhizal, animal parasitic, or saprotrophic). The user will thus obtain not only a taxonomic, but also a functional/ecological, fingerprint of the fungal community at hand. FUNGuild presently associates functional guilds at the family, genus, and species name levels, which presents a potential problem since these taxonomic ranks can include genera, species, or strains, respectively, with divergent ecological lifestyles. To address this issue, UNITE will include the assigned level of confidence provided by FUNGuild for each assignment to assist user interpretation. We also anticipate being able to associate assignments with individual DNA sequences (fungal individuals), such that a closer UNITE-FUNGuild collaboration—where all data are fed in both directions—would be able to provide highly detailed information on functional aspects of fungal communities to users of UNITE and FUNGuild alike.

The UNITE database is an open source, open access initiative driven by the mycological community. UNITE has a history of adapting to the needs and wants of the mycological community, and we mean to keep it that way. Any feature request forwarded to the UNITE staff will be considered for implementation, and all such implementations will be made freely available to the scientific community at large.
